# Supplementary material for: Information theoretic evidence for layer- and frequency-specific changes in cortical information processing under anesthesia
Source: PLoS Comput Biol. 2023 Jan 26;19(1):e1010380. doi: 10.1371/journal.pcbi.1010380 (PMC9904504; doi:10.1371/journal.pcbi.1010380)
Supplement: S1 Table — (PDF) [file pcbi.1010380.s001.pdf]

S1 Table. Priors of Bayesian linear regression

parameter	value
α	$Normal(\mu = 0, \sigma = 3)$
β	$Normal(\mu = 0, \sigma = 3)$
β_{sq}	$Normal(\mu = 0, \sigma = 3)$
σ	$HalfNormal\ (\sigma = 1)$
