# Supplementary material for: Information theoretic evidence for layer- and frequency-specific changes in cortical information processing under anesthesia
Source: PLoS Comput Biol. 2023 Jan 26;19(1):e1010380. doi: 10.1371/journal.pcbi.1010380 (PMC9904504; doi:10.1371/journal.pcbi.1010380)
Supplement: S2 Table — (PDF) [file pcbi.1010380.s002.pdf]

S2 Table. Results of LOO-CV model comparison for AIS in the time domain. In bold the model with the lowest LOO-CV score at each layer and brain area.

**model**	**LOO-CV score**
Infragranular PFC	-683.76 ±20.9
Infragranular PFC squared	**-674.74** ±20
Granular PFC	-899.4 ±20.69
Granular PFC squared	**-886.1** ±20
Supergranular PFC	-1070.8 ±16.9
Supergranular PFC squared	**-1070.24** ±17.4
Infragranular V1	-927.9 ±16.9
Infragranular V1 squared	**-721.7** ±24.34
Granular V1	-961.2 ±17.8
Granular V1 squared	**-799.56** ±21
Supergranular V1	-960.1 ±15.74
Supergranular V1 squared	**-786.6** ±16.5
