# Supplementary material for: Information theoretic evidence for layer- and frequency-specific changes in cortical information processing under anesthesia
Source: PLoS Comput Biol. 2023 Jan 26;19(1):e1010380. doi: 10.1371/journal.pcbi.1010380 (PMC9904504; doi:10.1371/journal.pcbi.1010380)
Supplement: S3 Table — (PDF) [file pcbi.1010380.s003.pdf]

S3 Table. Results of LOO-CV model comparison for AIS_{freq} at 62.5Hz -125Hz

model	LOO-CV score
Infragranular PFC	-1058.35 ±16.28
Infragranular PFC squared	**-571.62** ±19.31
Granular PFC	-831.4 ±15.1
Granular PFC squared	**-266.03** ±22.5
Supergranular PFC	-1027.40 ±49.34
Supergranular PFC squared	**-1017.10** ±50.52
Infragranular V1	-593.65 ±33.73
Infragranular V1 squared	**-593.21** ±34.61
Granular V1	-917.99 ±11.97
Granular V1 squared	**-562.23** ±20.56
Supergranular V1	-900.69 ±12.67
Supergranular V1 squared	**-359.86** ±27.3
