# Supplementary material for: Information theoretic evidence for layer- and frequency-specific changes in cortical information processing under anesthesia
Source: PLoS Comput Biol. 2023 Jan 26;19(1):e1010380. doi: 10.1371/journal.pcbi.1010380 (PMC9904504; doi:10.1371/journal.pcbi.1010380)
Supplement: S4 Table — (PDF) [file pcbi.1010380.s004.pdf]

S4 Table. Results of LOO-CV model comparison for AIS_{freq} at 31Hz -62Hz

**model**	**LOO-CV score**
Infragranular PFC	-876.19 ±18.22
Infragranular PFC squared	**-670.32** ±21.06
Granular PFC	-1053.19 ±33.38
Granular PFC squared	**-947.72** ±37.8
Supergranular PFC	-935.66 ±41.34
Supergranular PFC squared	**-929.95** ±42.21
Infragranular V1	-904.77 ±19.73
Infragranular V1 squared	**-849.49** ±20.27
Granular V1	-869.24 ±16.61
Granular V1 squared	**-863.24** ±16.79
Supergranular V1	-952.69 ±18.67
Supergranular V1 squared	**-890.80** ±21.50
