# Supplementary material for: Information theoretic evidence for layer- and frequency-specific changes in cortical information processing under anesthesia
Source: PLoS Comput Biol. 2023 Jan 26;19(1):e1010380. doi: 10.1371/journal.pcbi.1010380 (PMC9904504; doi:10.1371/journal.pcbi.1010380)
Supplement: S5 Table — (PDF) [file pcbi.1010380.s005.pdf]

S5 Table. Results of LOO-CV model comparison for AIS_{freq} at 15.6Hz -31.2Hz

**model**	**LOO-CV score**
Infragranular PFC	-607.85 ±22
Infragranular PFC squared	**-582.48** ±24
Granular PFC	-1056.38 ±39
Granular PFC squared	**-1021.56** ±40
Supergranular PFC	**-841.49** ±20
Supergranular PFC squared	-842.14 ±21
Infragranular V1	-949.41 ±39
Infragranular V1 squared	**-945.87** ±40
Granular V1	-870.78 ±44
Granular V1 squared	**-853.9** ±43
Supergranular V1	-922.84 ±30
Supergranular V1 squared	**-727.01** ±38
