# Supplementary material for: Information theoretic evidence for layer- and frequency-specific changes in cortical information processing under anesthesia
Source: PLoS Comput Biol. 2023 Jan 26;19(1):e1010380. doi: 10.1371/journal.pcbi.1010380 (PMC9904504; doi:10.1371/journal.pcbi.1010380)
Supplement: S6 Table — (PDF) [file pcbi.1010380.s006.pdf]

S6 Table. Results of LOO-CV model comparison for AIS_{freq} at 7.8Hz -15.6Hz

**model**	**LOO-CV score**
Infragranular PFC	-810.93 ±20
Infragranular PFC squared	**-723.32** ±20
Granular PFC	-1046.34 ±24
Granular PFC squared	**-986.50** ±25
Supergranular PFC	-1011.07 ±22
Supergranular PFC squared	**-988.49** ±20
Infragranular V1	-939.40 ±18
Infragranular V1 squared	**-837.64** ±18
Granular V1	-887.64 ±36
Granular V1 squared	**-853.82** ±35
Supergranular V1	-922.38 ±29
Supergranular V1 squared	**-706.28** ±38
