# Supplementary material for: Information theoretic evidence for layer- and frequency-specific changes in cortical information processing under anesthesia
Source: PLoS Comput Biol. 2023 Jan 26;19(1):e1010380. doi: 10.1371/journal.pcbi.1010380 (PMC9904504; doi:10.1371/journal.pcbi.1010380)
Supplement: S7 Table — (PDF) [file pcbi.1010380.s007.pdf]

S7 Table. Results of LOO-CV model comparison for AIS_{freq} at 4Hz -7.8Hz

**model**	**LOO-CV score**
Infragranular PFC	-859.47 ±22
Infragranular PFC squared	**-828.24** ±21
Granular PFC	-1056.88 ±20
Granular PFC squared	**-1042.70** ±20
Supergranular PFC	-999.75 ±19
Supergranular PFC squared	**-985.72** ±19
Infragranular V1	-958.89 ±21
Infragranular V1 squared	**-951.14** ±22
Granular V1	-887.64 ±36
Granular V1 squared	**-927.66** ±21
Supergranular V1	-955.66 ±21
Supergranular V1 squared	**-686.81** ±26
