# Supplementary material for: Information theoretic evidence for layer- and frequency-specific changes in cortical information processing under anesthesia
Source: PLoS Comput Biol. 2023 Jan 26;19(1):e1010380. doi: 10.1371/journal.pcbi.1010380 (PMC9904504; doi:10.1371/journal.pcbi.1010380)
Supplement: S8 Table — (PDF) [file pcbi.1010380.s008.pdf]

S8 Table. Results of LOO-CV model comparison for AIS_{freq} at 1.95Hz -4Hz

**model**	**LOO-CV score**
Infragranular PFC	-1073.27 ±21
Infragranular PFC squared	**-969.21** ±22
Granular PFC	-1059.54 ±21
Granular PFC squared	**-964.31** ±21
Supergranular PFC	-1073.08 ±20.68
Supergranular PFC squared	**-981.98** ±20
Infragranular V1	-963.49 ±30.52
Infragranular V1 squared	**-895.38** ±29
Granular V1	-963.14 ±27.24
Granular V1 squared	**-874.52** ±26
Supergranular V1	-956.37 ±21.26
Supergranular V1 squared	**-691.40** ±28
