# Supplementary material for: Information theoretic evidence for layer- and frequency-specific changes in cortical information processing under anesthesia
Source: PLoS Comput Biol. 2023 Jan 26;19(1):e1010380. doi: 10.1371/journal.pcbi.1010380 (PMC9904504; doi:10.1371/journal.pcbi.1010380)
Supplement: S9 Table — (PDF) [file pcbi.1010380.s009.pdf]

S9 Table. Results of LOO-CV model comparison for AIS_{freq} at 0.9Hz -1.9Hz

**model**	**LOO-CV score**
Infragranular PFC	-889.0 ±25
Infragranular PFC squared	**-813.46** ±27
Granular PFC	-915.27 ±23
Granular PFC squared	**-854.14** ±24
Supergranular PFC	-993.8 ±25
Supergranular PFC squared	**-923.4** ±25
Infragranular V1	-959.4 ±39
Infragranular V1 squared	**-939.76** ±37
Granular V1	-919.5 ±30
Granular V1 squared	**-881.5** ±28
Supergranular V1	-875.3 ±16
Supergranular V1 squared	**-822.6** ±18
