# Supplementary figures and images for: Information theoretic evidence for layer- and frequency-specific changes in cortical information processing under anesthesia
Source: PLoS Comput Biol. 2023 Jan 26;19(1):e1010380. doi: 10.1371/journal.pcbi.1010380 (PMC9904504; doi:10.1371/journal.pcbi.1010380)

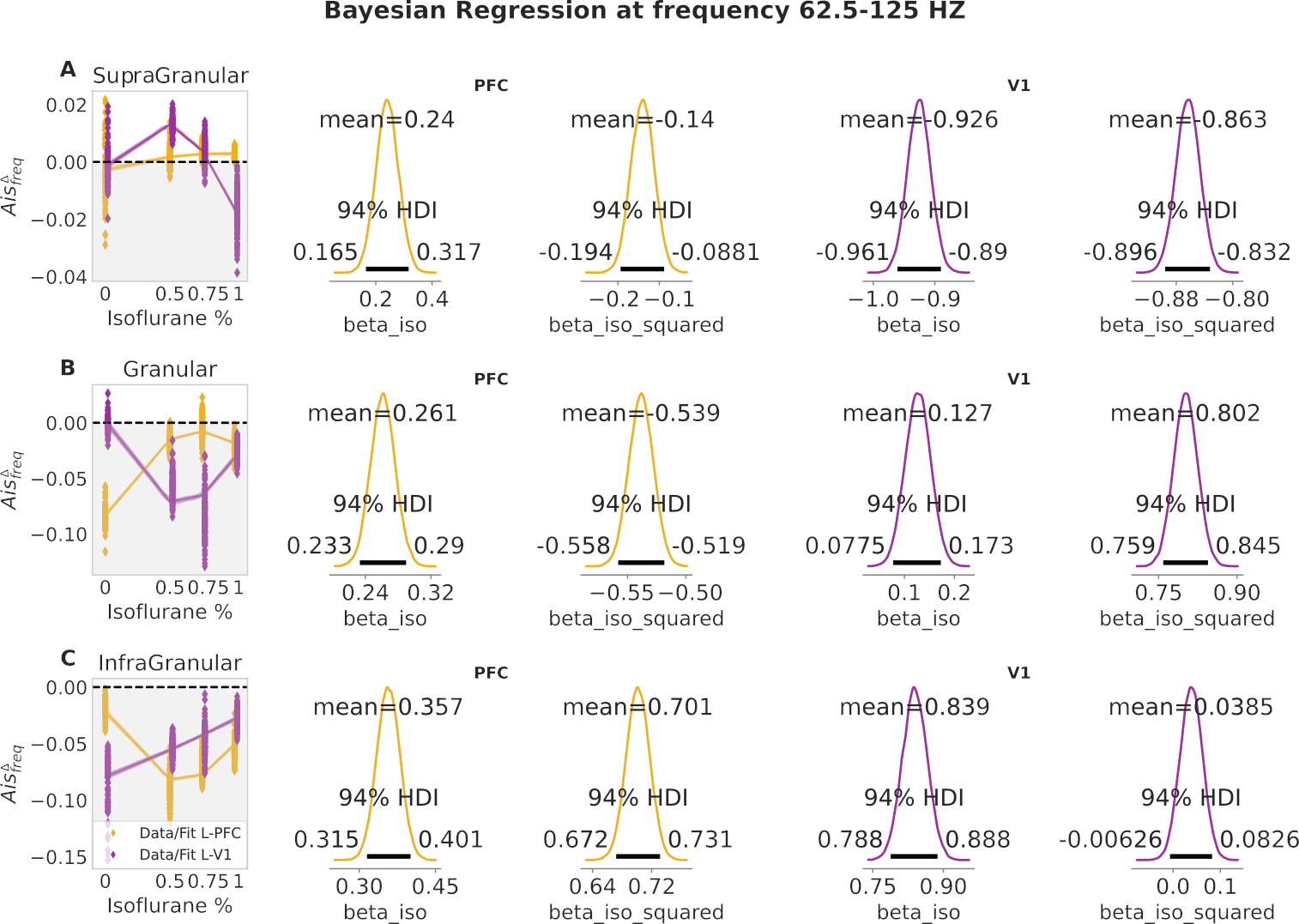

Supplement: S1 Fig — Panel A, left, Bayesian regression fits for supragranular layer at PFC (yellow) and V1 (purple). Panel B, left, Bayesian regression fits for granular layer at PFC (yellow) and V1 (purple). Panel C, left, Bayesian regression fits for infragranular layer at PFC (yellow) and V1 (purple). Middle columns, posterior mean for beta iso and beta iso squared coefficients at PFC site, for panel A, B and C. Right columns, posterior mean for beta iso and beta iso squared coefficients at V1 site. Shaded area in the regression fits represents 94% HDI. Shaded gray background for AISfreqΔ values that are below zero (i.e. no frequency specific drop). (TIFF) [file pcbi.1010380.s010.tiff]

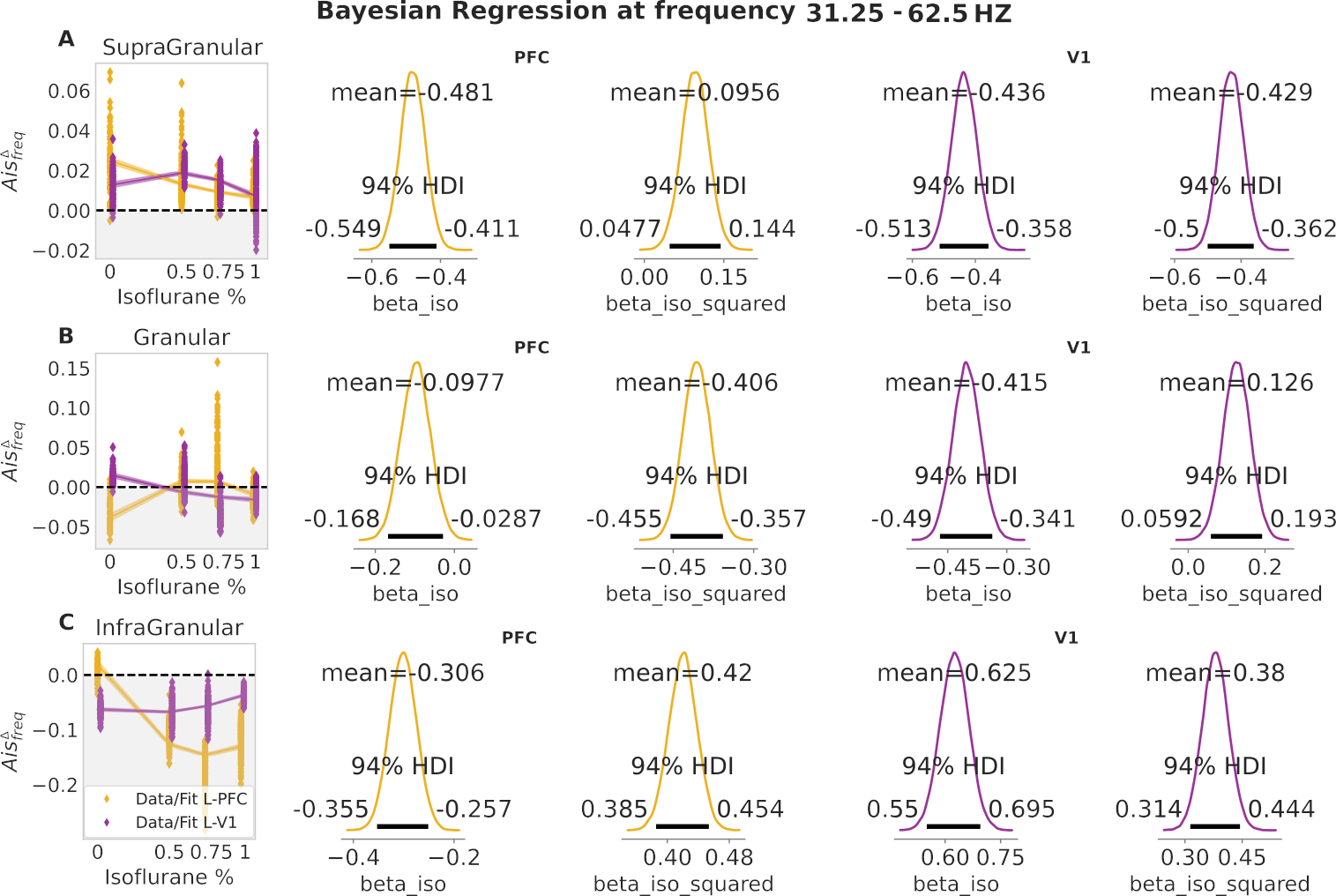

Supplement: S2 Fig — See S1 Fig for display conventions. (TIFF) [file pcbi.1010380.s011.tiff]

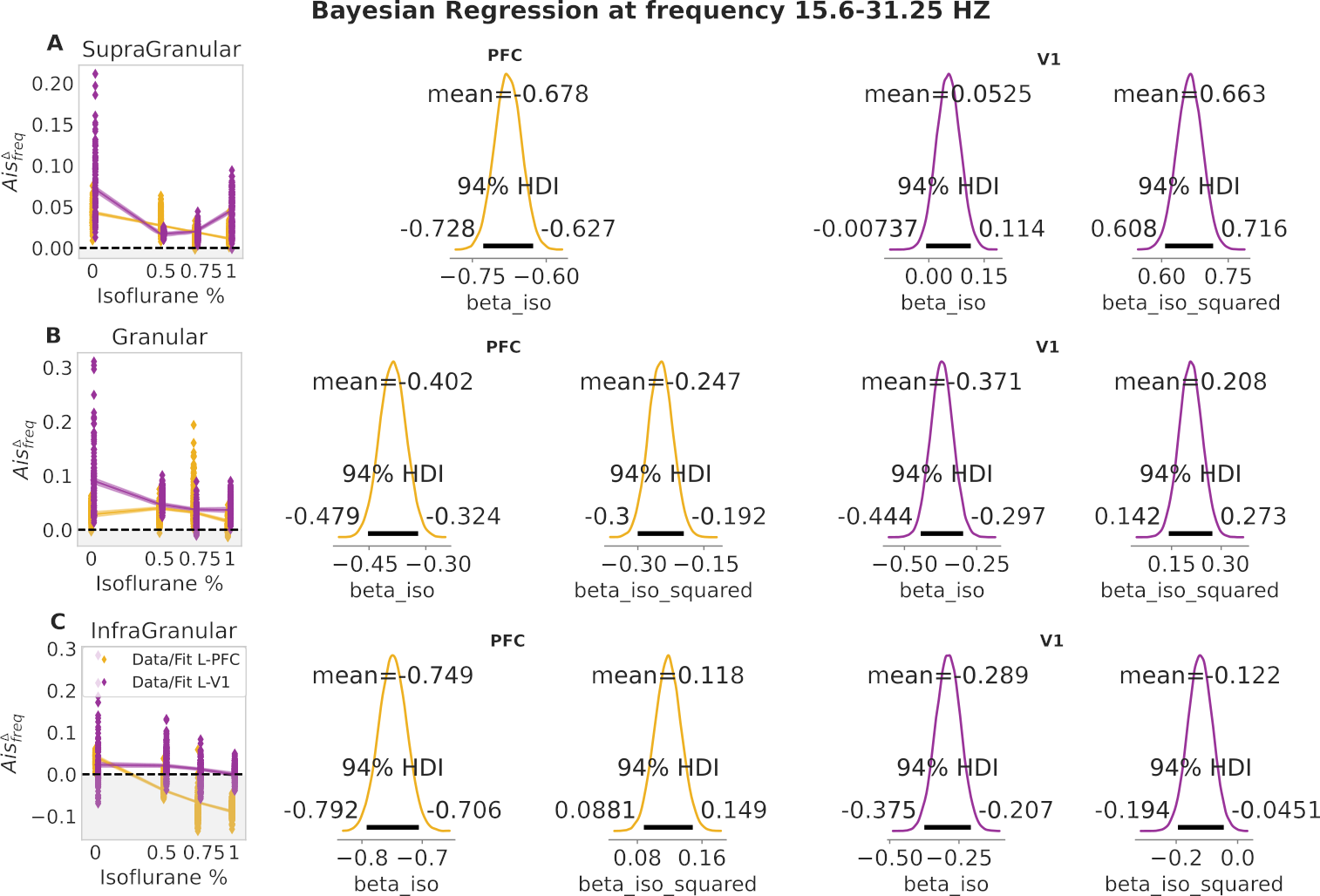

Supplement: S3 Fig — See S1 Fig for display conventions. (TIFF) [file pcbi.1010380.s012.tiff]

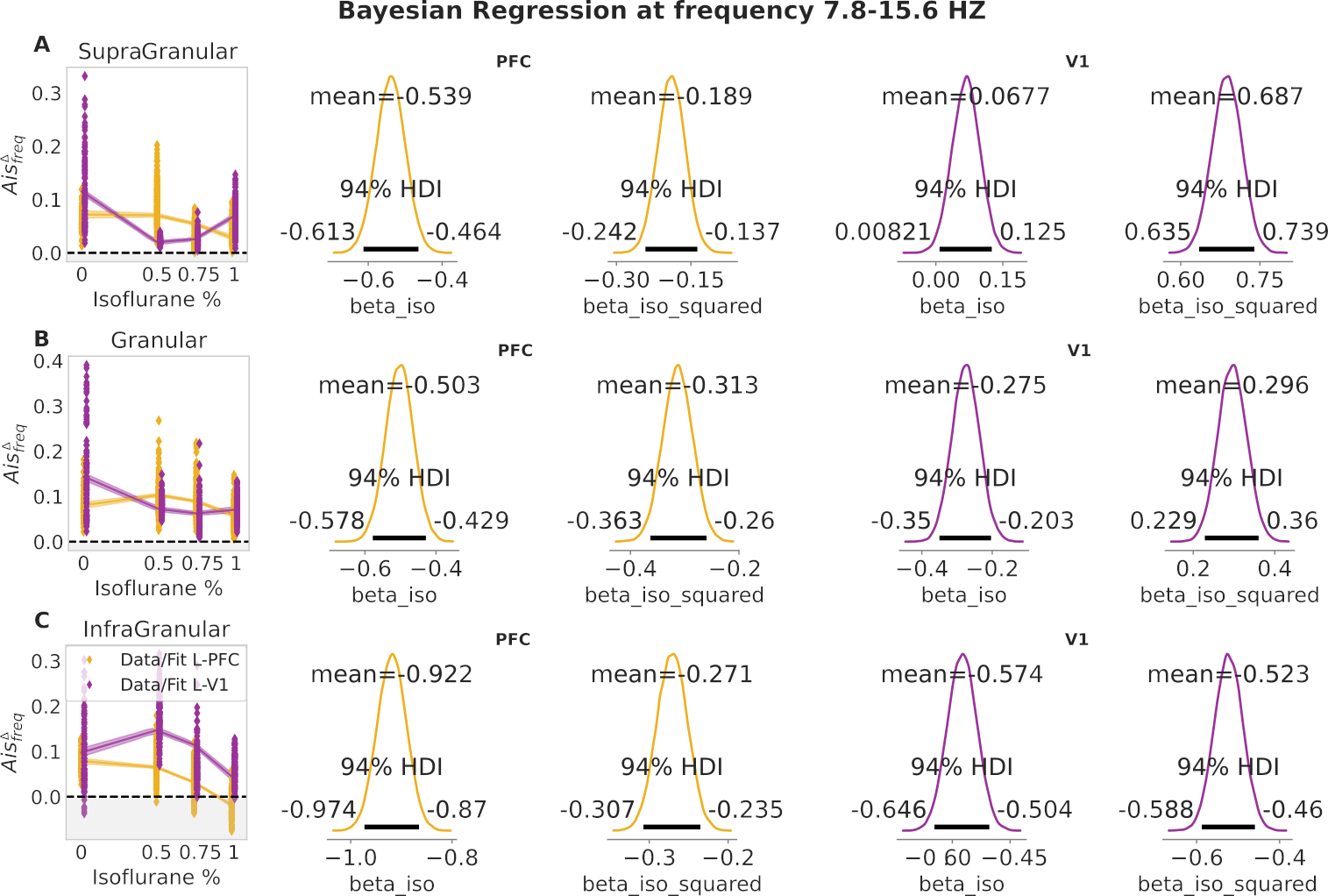

Supplement: S4 Fig — See S1 Fig for display conventions. (TIFF) [file pcbi.1010380.s013.tiff]

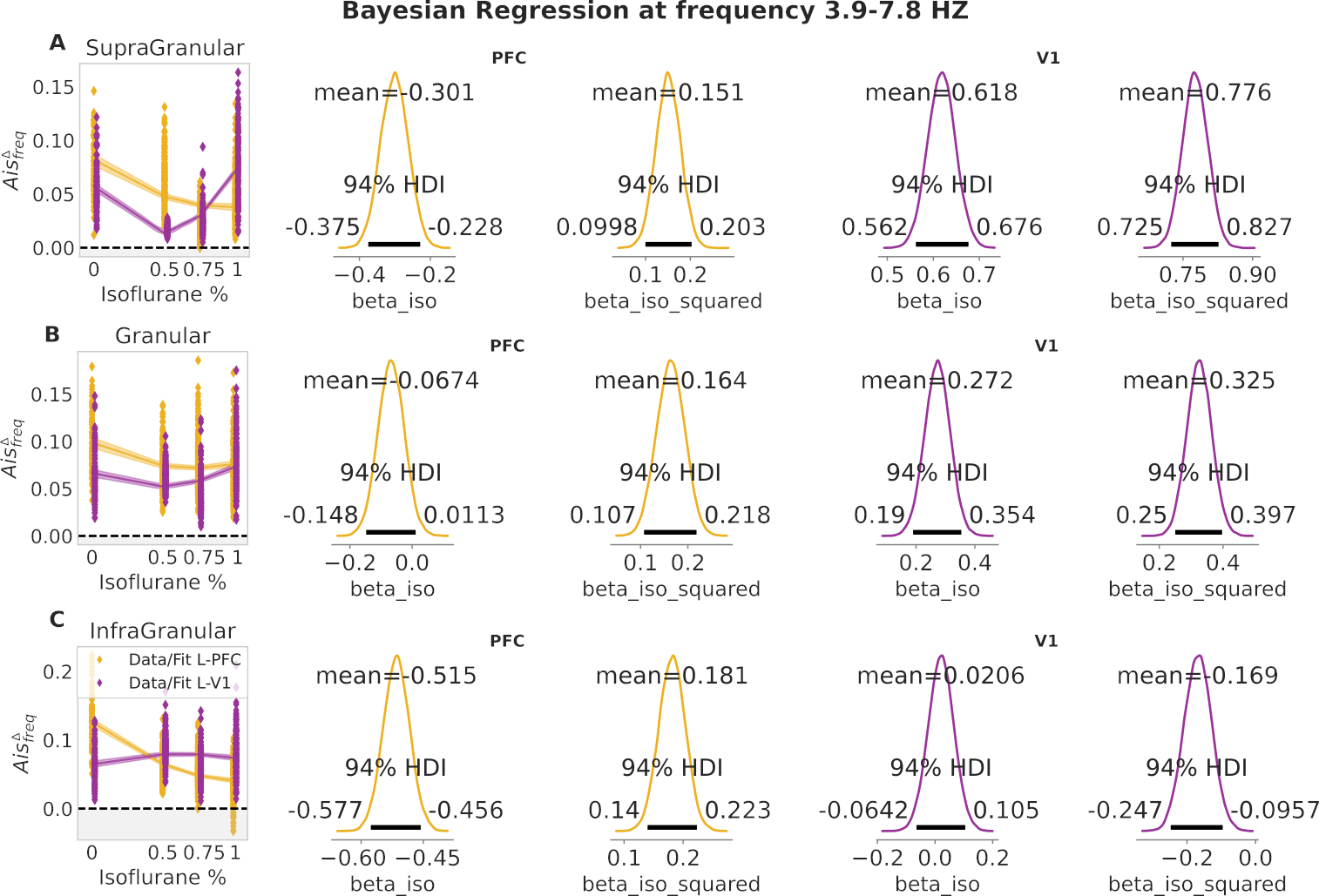

Supplement: S5 Fig — See S1 Fig for display conventions. (TIFF) [file pcbi.1010380.s014.tiff]

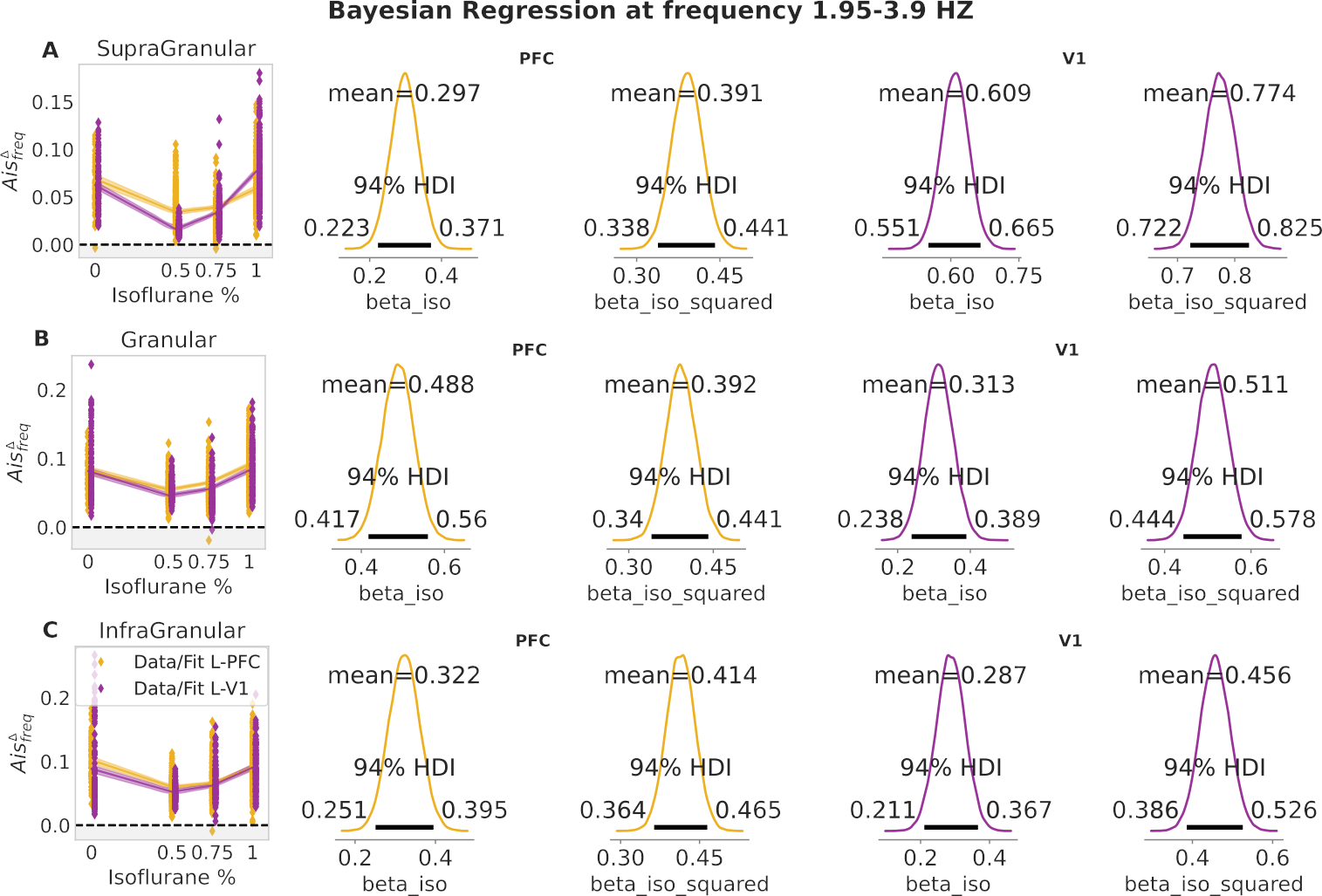

Supplement: S6 Fig — See S1 Fig for display conventions. (TIFF) [file pcbi.1010380.s015.tiff]

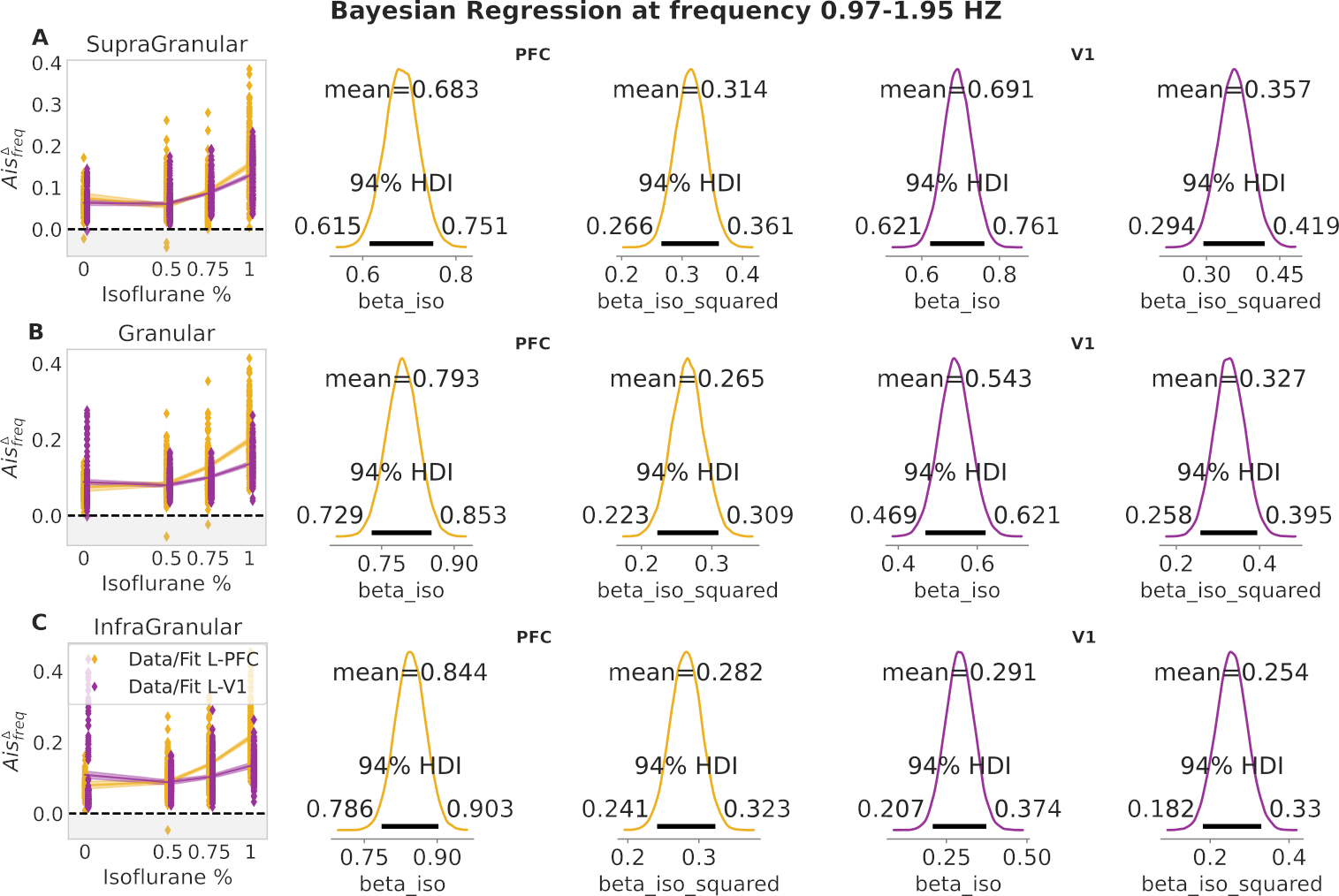

Supplement: S7 Fig — See S1 Fig for display conventions. (TIFF) [file pcbi.1010380.s016.tiff]

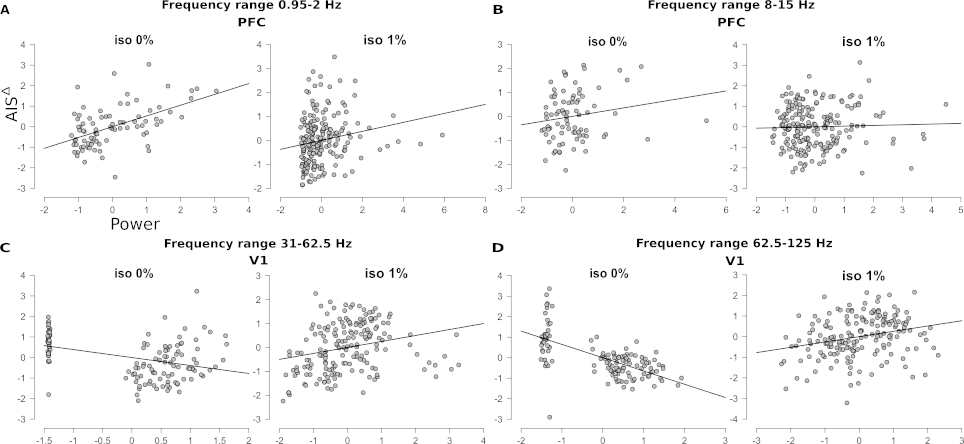

Supplement: S8 Fig — Bayesian correlation of spectral AIS with spectral power in different frequency ranges. (TIFF) [file pcbi.1010380.s017.tiff]

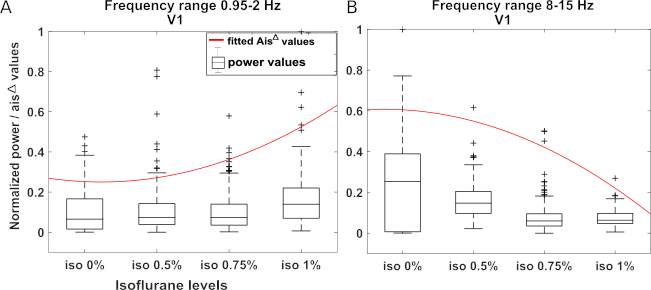

Supplement: S9 Fig — Panel A, the modulation of AIS (red curve) and spectral power at isoflurane levels, in the delta band (black box-plot). Panel B, the modulation of AIS (red curve) and spectral power at isoflurane levels, in the alpha band (black box-plot). (TIFF) [file pcbi.1010380.s018.tiff]

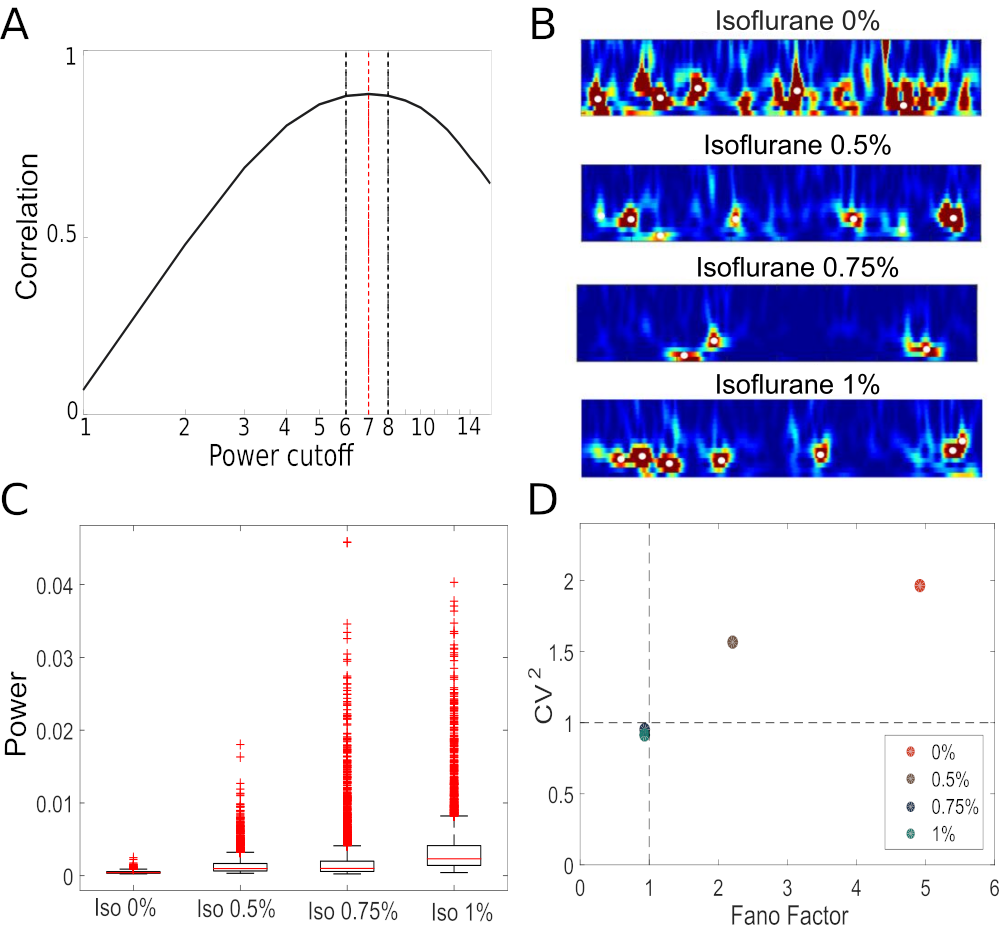

Supplement: S10 Fig — Panel A, Pearson’s correlation coefficient between mean alpha/beta power and the percent of pixels in the spectrogram above cutoff in the non-averaged spectrogram. Various median cutoffs were calculated and plotted on a log scale. Black line correlation is the mean across isoflurane concentrations. Panel B, example of alpha/beta burst detection for each isoflurane concentration (single trial). White dot denotes the local maxima in the spectrogram, with maxima power above 7x median power cutoff. Panel C, Boxplot denotes the mean frequency power at the burst maxima, for different isoflurane concentrations. Panel D, Fano Factor and CV2 for each isoflurane concentrations. (TIFF) [file pcbi.1010380.s019.tiff]
