# Supplementary material for: Information theoretic evidence for layer- and frequency-specific changes in cortical information processing under anesthesia
Source: PLoS Comput Biol. 2023 Jan 26;19(1):e1010380. doi: 10.1371/journal.pcbi.1010380 (PMC9904504; doi:10.1371/journal.pcbi.1010380)
Supplement: S1 Text — (PDF) [file pcbi.1010380.s020.pdf]

S1 Text. Alpha-Beta bursts analysis pipeline. For identification of the alpha/beta burst in the non-averaged spectrogram we employed the method adopted in [1]. At first, the alpha/beta burst events were defined as the local maxima in the trial time-frequency analysis matrix for which the power exceeded a set cutoff (see S10 Fig, panel B). To choose the power threshold cutoff, we computed the correlation between the percent area above cutoff in the spectrogram (i.e. pixels in the spectrogram) and the mean power in the frequency range $(8 - 15Hz)$. The average correlation across isoflurane concentrations was highest at 7x median power (see S10 Fig, panel A). Secondly, we aimed to quantify if the underlying generation mechanism of the burst events followed a Poisson process at high isofurane concentrations. Thus, we calculated the inter-event-interval (IEI) as the time difference between two consecutive events and the trial-to-trial number of events per trial (EpT). Then, we computed the coefficient of variation of the IEI as:

$$CV^2 = \frac{Var[IEI]}{E[IEI]^2} \tag{15}$$

where Var is the variance and E is the mean and the Fano Factor, to quantify the variability of the EpT as:

$$FF = \frac{Var[EpT]}{E[EpT]} \tag{16}$$

For a Poisson process the Fano Factor and the CV^2 would be 1, with both factors having a similar value. Values of the Coefficient of variation and the Fano Factor greater than 1 indicates high variability and deviation from independence Poisson spiking (see S10 Fig, panel D).

References

1. Shin H, Law R, Tsutsui S, Moore CI, Jones SR. The rate of transient beta frequency events predicts behavior across tasks and species. eLife. 2017 nov;6:e29086. Available from: https://doi.org/10.7554/eLife.29086.
