# Supplementary material for: Information theoretic evidence for layer- and frequency-specific changes in cortical information processing under anesthesia
Source: PLoS Comput Biol. 2023 Jan 26;19(1):e1010380. doi: 10.1371/journal.pcbi.1010380 (PMC9904504; doi:10.1371/journal.pcbi.1010380)
Supplement: Attachment — Submitted filename: response_to_reviewers.pdf [file pcbi.1010380.s021.pdf]

Leibniz-Institut für Resilienzforschung

Dear reviewers,

We thank the reviewers for the careful reading of the manuscript to which we reply point by point below. We hope to have addressed all their concerns and think that the manuscript has strongly improved based on their valuable input. Most importantly we now more closely link the results obtained by our spectral AIS analysis to theories of cortical computation on the one side and to the neurophysiology of anesthesia on the other side. Note that Figure 11 (which now became Figure 6) had the raw difference between the AIS of the original and the surrogate data, with negative values indicating AIS in that figure. The overall convention of the manuscript however was and is to report this difference in a way such that positive values indicate the presence of AIS. We have now redone the figure in such a way that it conforms with the overall convention.

Yours sincerely on behalf of all co-authors,
Edoardo Pinzuti

Point by point replies:

Reviewer 1: Summary The article by Pinzuti et al. proposes an approach to assess frequency-specific storage of information in neural systems, with application to cortical information processing under anesthesia. The method builds on previous work of the same group in the context of (i) estimating active information storage (AIS) [e.g., Ref. 7] and (ii) estimating frequency-specific information transfer using wavelet-transform surrogate data [Ref. 10]. Despite this incremental aspect, the proposed methodology is original as it fills a gap in the current literature in the field, and its application showing the effects of anesthesia on neural information processing convincingly shows the usefulness of the approach (e.g., when comparing frequency-specific AIS to time-domain AIS and to spectral power). In view of this, and considering that the neurophysiological findings are original and the paper is well written, I recommend acceptance of the manuscript after addressing the comments in the following.

General points:

- Recent studies have shown that the two other components of information processing, i.e. information transfer and information modification, in the particular case of linear Gaussian processes, can be formulated analytically in the frequency domain and related to their time-domain counterparts in an intuitive way [e.g., Chicharro, D. (2011), Biological cybernetics, 105(5), 331-347; Faes, L., et al. (2021, Philosophical Transactions of the Royal Society A, 379(2212), 20200250; Antonacci, Y., et al. (2021), IEEE Access, 9, 149486-149505]. These works are relevant to the present paper, because similar formulations could be provided also for the information storage, and therefore it would be interesting to compare them, at the level of the discussion, with the approach proposed here and in Ref. [10] to assess frequency-specific information storage and information transfer.

Reply: We thank the reviewer for pointing out these interesting references Gaussian processes, we have now added them in the subsection: Relation to previous approaches.

- Also in view of the previous comments, the Authors could mention in the subsection about "Future directions" the extension of their method to the evaluation of information modification, also highlighting the potential issues related to its implementation.

Reply: We have now discussed the possible extension and related issues to the information modification in the subsection: Future Directions.

- Another aspect that could be mentioned in the discussion is that the measure of information storage, computed in a model-free way as done in this work, reflects also the nonlinear interactions between rhythms manifested in a signal at different frequencies; therefore, destroying a specific time scale as is done with the surrogates would eliminate such interactions. This effect could be viewed as a way to investigate the presence of nonlinear dynamics related to the cross-talk among different frequencies.

Reply: We have now added this point in the subsection:On the possibility of cross-spectral information storage.

- Figures 3-10 are clear and explicative, but appear not very informative if taken individually, and their repetitive style may make reading the main manuscript heavier. I would suggest to move these figure in the supplementary material, and generate 2-3 figures in the main paper where the same results are presented in a more compact representation.

Reply: We agree with the reviewer that Figures 3-10 were too many. Now, we have created two new figures, Figure 4 and Figure 5. For Figure 4 we have condensed all frequencies ranges to one figure. We think that the Bayesian regression fit contains satisfactory information about the analysis with the estimated mean of the posterior distribution and the 94% high density interval (HDI), or posterior credible interval. In Figure 5 we made a selection (based on the Result section) of the Bayesian posterior distributions that were of particular interest. All the previous figures have been moved to the Supporting Information and can be consulted by the reader, if necessary.

- Eq. (8) describing the AR(2) process seems to be incorrect (p^2 should be multiplied by S_0(t-2), and an additive noise term W(t) is missing). Moreover, although it is stated that the process exhibits long-term memory, this does not appear from Eq. (8) where only short-term dependencies are modeled.

Reply: We thank the reviewer for having spotted the mistake, indeed an AR(2) process does not have long-term dependencies. We have now reported the correct simulations, i.e. a simulation of a Fractionally integrated AR process. The combination of an AR process with the fractionally integrated white noise can exhibit stochastic oscillations and long-memory. Since many applications of AIS and spectral AIS are on time-series with longer-term memory dynamics, we believe that such simulation fits better as a demonstration of the current method.

- The citation of Ref. [34] is wrong/incomplete (e.g. journal name)

Reply: We thank the reviewer for catching this referencing error, we have now pointed to the correct journal.

- Page 10: a couple of sentences describing how the data were collected and pre-processed would be useful.

Reply: We have now added a subsection: Electrophysiological recordings and pre-processing

Minor points/typos:

- Page 6, "Fig. ??"
- Page 9, line 5 after Eq. (9) "Fig 2, A" should be "Fig 2, panel C"
- Page 12, "Bayesian model comparison ??"
- Page 17, a question mark should be put at the end of the question 2 in the first paragraph of the Discussion
- Page 17, "asses frequency"

Reply: We thank the reviewer for catching these errors.

Reviewer 2: Summary This is potentially a very interesting work on the intersection of information processing, the role of cortical layers and frequency bands, with conscious states. Although I feel not qualified to review the mathematical parts of this manuscript, I believe the findings could provide new avenues for understanding (un)consciousness states induced with anaesthesia. However, in order to do so, I think the following points should be clarified or emphasized.

General points:

- The authors introduce very little about the role of different cortical layers on frequencies/ information processing. This is merely stated that there are different effects across the different cortical layers, but as the main aim of the study seems to be the evaluation of the interaction between information processing, cortical layers and frequency bands, I would appreciate a more in-depth introduction of previous findings (i.e., at least including the main results of previous work). Likewise, the (minimal) differences in the findings for PFC and V1 electrodes are discussed, but the rationale for placing electrodes in these cortical areas specifically is not highlighted. It is clear that the data has been previously acquired, yet it would be good to emphasize in the introduction the logic for including/contrasting these two cortical areas based on their different (functional) cortical organization.

Reply: We have now added a more in depth description of the role of cortical layers in terms of information processing as well as the reason why the ferret model is a good model to obtain invasive LFP recordings in the last part of the Introduction.

- The material and methods section does not include sufficient information about the animal model used in the current work. A reference is provided for more information, but I encourage the authors to provide at least basic information regarding the number of recording sites per ferret (i.e., number of electrodes and number of recording sites per electrode), the isoflurane dose (and how the different doses relate to behaviour/consciousness), the determination of the point of LOC in the ferrets, the duration of the recording. Furthermore, no mention of the data pre-processing and dropout are provided. A comprehensive section should be included in the manuscript to ease interpretation of the results.

Reply: We have now added a subsection: Electrophysiological recordings and pre-processing

- A common finding in different conditions of altered consciousness, consistent in various species, is a general slowing of the observed oscillatory activity. This has also been studied in reference 15 (in the same dataset), but no overview of these results is provided in the manuscript. It could help to place the results on AIS in context, as the reader might wonder how the results on AIS depend on potential shifts in the spectral power peaks specifically, rather than a more mechanistic role of the different cortical layers and feedforward/feedback connections. This relation between AIS and spectral power is partially addressed in Figure 11, but only for very specific delta and alpha bands, and does not account for any potential shifts in spectral power peaks. Also, do the authors have an explanation for the larger variability in normalized power at higher isoflurane doses?

Reply: We have extended Figure 11 (became Figure 6) now to show more different frequency bands and discuss the relation of AIS findings to spectral power changes, and especially to the prominent high-power low frequency and neurophysiology in more detail in the rewritten discussion section. With respect to the larger variability of power at higher isofluorane doses we computed the Fano factor across the 8s segments from which power values were computed and can indeed confirm that it goes up, although not dramatically. On the one hand this means that variability goes up simply because spectral power goes up, but on the other hand there is indeed an additional source of variability. However, we would like to not speculate about it's origin. If the reviewer has a specific hypothesis here, we are of course willing to consider/cite it.

- In the manuscript it is stated that the mechanism of action of different anaesthetics is unknown. This is correct, the full mechanism of action of (here) isoflurane is unknown, but we do know that it acts on the GABAa receptor, glutamate receptor and alters cellular channel properties. A discussion about how these different properties could be related to alternation in information processing could help to explore the relation to (un)conscious states.

Reply: We have now added this information in the Discussion session. We outlined the effect of isoflurane on GABAa receptors and thalamus with a possible cellular mechanism of the disruption of cortico-cortical and thalamic-cortical feedback, based on the work of Suzuki et al. [58].

- I believe the text would become more reader friendly if the literature is referred to in a more direct fashion. E.g., in the results: "However, previous work on spectral power [15, 16], as mentioned above, showed that anesthesia modulates cortical areas and layers differently.", then it would be nice to read how exactly it differed. In the discussion: "Even though the molecular targets of anesthetic agents are well known [42],", then I suggest the authors to at least mention the targets of isoflurane.

Reply: We considerable rewrote the discussion section, and also tried to incorporate the missing details on isoflurane action

Minor comments:

- -Please mention in the abstract that this is a study in ferrets, not in human.

Reply: we have added the information to the abstract

- -The axes of Figure 11D refer to delta, the text refers to alpha.

Reply: We have changed the word AISDelta of the Figure 6 (Fig 11 became Fig 6) y-axis to $AIS^{\triangle}$ to avoid confusion between the indication of an AIS difference and the Delta-frequency band. Indeed, the $AIS^{\triangle}$ refers to the difference between the AIS found in the time domain and the AIS estimated in the spectral domain at specific scale/band (in the Figure 6, panel E in the range 0.9-2Hz 'delta band' while in Figure 6, panel F in the range 8-15Hz 'alpha band'). We apologize for the confusion.

- Author summary missed a word: Here -we- introduce such a measure and study how isoflurane anesthesiaaffects the local information processing in the ferret prefrontal and primary visual areasaround loss of consciousness.
- Discussion misses a citation at "only a handful of studies exist to date [?,6-8, 13],"
- Discussion misses a word: where activity -in- supragranular layers
- Hz is sometimes written italic and sometimes not.

Reply: We thank the reviewer for catching these errors.

Reviewer 3: Summary In this paper, Pinzuti et al. tackle the problem of forming a spectrally resolved estimator of active information storage (AIS), and using it to characterise some aspects of neural computation in data from ferrets undergoing general anaesthesia. The authors are able to formulate a spectral AIS estimator thanks to a direct application of a previous method, and apply it to a rich dataset of LFP recordings across cortical regions and layers. The explicit focus on linking information processing with features of neural activity (oscillations) is interesting; and I commend the authors for running their statistical analysis with the powerful pymc3 library as well as for their use of Bayes factors for analysis.Overall, I find the subject of the paper very interesting and worth pursuing. However, as it stands, the method isn't particularly novel by itself, and a clearer presentation and interpretation of the results is sorely lacking. For these reasons, and despite the great potential of this line of work, I cannot recommend the paper for publication without substantial revision.

General points:

- 1) Results and interpretation. The results of the AIS analysis of ferret LFP data are spread across a lengthy description and 8 figures. The results are described in great detail, but without any clear summary or clear interpretation of what they mean for the neuroscience of anaesthesia (or the neuroscience of consciousness more generally). With a few minor exceptions, the authors rarely draw specific conclusions showing how the spectral AIS yields new insights about cortical dynamics under anaesthesia, and rely on rather vague statements (e.g. alluding to the "different computational properties of the layers" [p.3], but without specifying what these properties might be).This makes it really hard for the reader to build a coherent picture of the results, other than "results varied widely between bands" and "lower frequencies are more spatially homogeneous." To be clear: I don't mean to say that the results are meaningless or can't be interpreted; I mean that with the current presentation it's extremely difficult for the reader to make sense of so much information.

Reply: We agree with the reviewer that Figures 3-10 were too many, we have now created two new figures (Figure 4 and 5). Figure 4 contains the regression fit for all frequency band while Figure 5 a selection of the Bayesian posterior distribution relevant for the results description. Old Figures 3-10 are now moved to the Supporting Information and can be consulted by the reader, if necessary.
We have placed our study in the context of the recent developments in anesthesia research. For this, we rewrote the section named "Modulation of spectral information storage according to distinct functional roles across cortical layers by anesthesia"

- I would strongly encourage the authors to make a smaller number of more representative figures that can convey the results in a more convincing way. While I am a fan of detailed statistical reporting, I see no reason why all ~ 90 posteriors should be in the main text instead of in the supplementary material.

Reply: See previous answer for the new arrangement of the figures.

- A clearly presented analysis of spectral AIS and its relationship to anaesthesia/consciousness in the different layers and regions, accompanied by a strong theoretical argument or perhaps even a computational model, would greatly strengthen the paper.

Reply: We have now linked our results to a theoretical account of anesthesia effects based on predictive coding theory and present a consistent interpretation of frequency-specific changes of AIS under Anesthesia. A full computational model however, is beyond the scope of this empirical study.

- 2) Meaning of the test. Overall, the statistical properties of the spectral AIS are a bit unclear. The examples are very useful in showcasing spectral AIS in systems with clear narrow-band activity, but this leaves many questions unanswered:- What is precisely the null hypothesis behind the null distribution? Can it be formalised mathematically?

Reply: The null hypothesis tested by the approach is: " The wavelet-coefficients of the frequency component of interest in the process time-series are exchangeable in their temporal relation to the future of the process (Y_t) with respect to the mutual information present in the past of the process ($Y_{<t}$) about the future (Y_t)"

- The authors demonstrate the test is sensitive to information storage in a particular band, by showing that it successfully identifies processes with activity in the band of interest. However, they do not show that the test is specific, which they should do by showing the test finds no significant effects in a system with AIS in all bands except the band of interest. (Or, even better, in a system with high spectral power but no AIS in the target frequency band, if this is possible, which would very strongly emphasise the authors' point that spectral AIS is meaningfully different from power.)

Reply: We have now performed the following analysis to test the specificity of the method. We have generated a signal consistent of two sinusoids at 50Hz and 12 Hz and additional

noise. The spectral AIS analysis show no significant spectral AIS at all scales not corresponding to these frequencies, and so demonstrating the specificity of the test. We repeated this analysis 500 times to asses that the false positive results are below the alpha level of 0.05. This analysis is now included in the section Results (see Figure 2 panel B).

- In Eq. 7, why is the future state taken from the unshuffled process?

Reply:The objective of the proposed method is to estimate the spectral contribution of the past state of a process to its current value. To this end, we need to shuffle only the past state of the process ($Y_{t,n}$), keeping the future of the process (Y_n) unshuffled. In cases where one might want to investigate a possible non-linear mechanism by which for example low frequencies in the past of a process are transformed to high frequency in the future of the same process then also Y_n can be shuffled. This is already possible in our implementation with the setting *spectral analysis type* $='$ *both*$'$, where 'both' refers to shuffling the past and the future of the process under analysis. We made a description of this parameter setting in the demo script, and mention this possibility in the discussion, in subsection "On the possibility of cross-spectral information storage".

- Perhaps most importantly, what is the meaning of surrogates having higher AIS than the original signals? These cases are clearly very significant (e.g. in Fig. 1), yet they are almost completely left unmentioned in the text, with the exception of some unproven statements that "only positive values correspond to a significant contribution to the formation of AIS."

Reply: First, we would like to mention, that there is no significant AIS in the time domain, and, therefore, time-series of this kind would not enter our spectral analysis in real-world analyses. However, in the current manuscript we specifically submit such time-series to an analysis by our spectral AIS-algorithm to show that we do not find false positive results in this case.
The effect the reviewer observed (i.e. increased AIS in the surrogate data in certain bands) well known (e.g. Pinzuti 2020, and reference therein), and is indeed one of the core motivations to shift all spectral processing to the creation of surrogate data. This is because spectral operations on data (e.g. filtering etc.) often have the negative side effect of introduction unwanted temporal correlations - which would lead to false positives when applied to the original data of interest, but only to more conservative statistical behaviour when used on the side of surrogate data (remember that we are looking for a significant drop in the surrogate data-based distribution of AIS values).
This problem has been also discussed in the context of spectral mTE analyses in some more detail in Pinzuti 2020. In the specific case the reviewer refers to the distorting effect in action for scales/frequencies $30 - 60Hz$. In fact when analysing the surrogate data time series created by the shuffling of wavelet coefficients, we find an increase in lagged autocorrelation which here explains the AIS effects (Note, however that AIS is not merely linear, lagged auto-correlation). As it can be seem in Figure 1 panel A, the increased AIS occurred only sometimes when the sample that showed artificial increased lagged correlation enter the spectral AIS computation. We have, therefore, included a more detailed explanation in the results section — i.e. immediately where these results are presented.

- In multiple places (e.g. page 18) the authors refer to the proposed method as a frequency decomposition. But, in what sense is it a decomposition? It doesn't seem to me that AIS_{freq} meaningfully decomposes the time-domain AIS (compare to e.g. PID, which can be called a decomposition because all atoms sum up to the quantity that is being decomposed, namely the joint mutual information).

Reply: We changed the term "spectral decomposition" at page 18 with "spectrally resolved AIS". Also we changed "MODWT decomposition" and "wavelet decomposition" into "MODWT transform" and "wavelet transform" . In the subsection Future directions we introduced also the possibility of having a spectral resolved PID, in this case we still used the word decomposition in the context of PID in the sense expressed by the reviewer.

- Finally, the comparisons between spectral power and spectral AIS were very interesting, and a crucial part of the authors' argument that spectral AIS reveals new information that was not available with previous tools. Unfortunately, this subsection is the shortest one in the Results section, and these relationships between spectral AIS and other methods were not investigated for the three example systems.

Reply: We have now added additional analyses to more clearly demonstrate the added value. We simulated three signals:

- **A signal with randomly spaced burst of activity and random phases**
- **A single high burst at the end of the signal**
- **A sinusoid with gaussian noise.**

We repeated this simulation 100 times and scaled the signals to obtain similar median power. We expected to see that the random burst and single burst would have much lower AIS and spectral AIS compared to the sinusoid, even if the power is similar. Indeed, the result showed correctly that independently of the power -which is roughly equal- , the AIS and spectral AIS can have a different profile across these signals. Additionally, we performed an alpha-beta burst analysis and showed that the decreased AIS might be related to the fact that the underlying burst generating mechanism becomes like a Poisson process.

- I would encourage the authors to clarify the statistical properties of their estimator (especially the issue of surrogates having higher AIS), and explore more systematically the relationship with spectral power.

Reply: see previous answers for these points

- 3) Novelty The method used in this paper is a direct application of a previously published method by the authors in this same journal. Substantial fractions of the text and Figure 1 are directly adapted from this previous paper. The data has also been analysed multiple times in previous publications, including a paper by some of the authors using AIS, as well as another one using the authors' spectrally resolved transfer entropy (TE) measure. Therefore, the potential for novel contributions of the paper is rather limited.

Reply: While the data has been already analyzed in term of power and MU activity, this is the first analysis of such data across cortical layers with respect to the spectral AIS. Furthermore, if Gaussian and linear assumptions of the data can not be made, this seems to be the only method available to compute AIS in the frequency domain (that also detects non-linear dynamic). The method goes in the direction of having TE, AIS and PID (see "Future directions" for a discussion about it) also in the frequency domain for non-gaussian data.

While I do not see this as a critical flaw for publication, it does mean that the authors should do significant additional work to satisfy major comments 1 and 2 above and clearly show the novel contributions of this manuscript.

Minor comments:

- The paper has several minor mistakes and typos throughout that should be addressed. As a non-exhaustive list of examples: p. 16, "frequencies range" (should be "frequency ranges"); p. 18, "Hz" is inconsistently written both with and without italics; p. 14, undefined reference; p. 16 "as the power increase" (should be "as the power increases"); etc).

Reply: We thank the reviewer for catching these errors.

- I find the notation is at times inconsistent. For example, N is used to denote both the length of the time series and the number of surrogates. In page 7, it seems W_{J_0} represents all wavelet coefficients, while from the definition in page 5 it would seem it is only the coefficients for the J_0 scale. Vector notation is also inconsistent, as Y is written both with and without bold font in page 4, and X is written without bold font. Specifically on the topic of vector notation, I think it would be much clearer for the reader if the authors were to specify the size of all matrices and vectors involved as they are introduced.

Reply: We thank the reviewer for spotting this duplicate use of the symbol N. We have now changed the notation. For surrogates we use "S", where 'S' is set to a number based on the desired critical level for statistical significance and the algorithm is repeated for s=1,...,S. We chose S to be consistent with the work of Novelli 2019, which presented the statistical step to compute the embedding of Y for AIS in the time-domain. With respect to the use of Vectors being indicated by bold font, we have now checked the manuscript that all vectors are bold font, all individual random variables and their realization are regular font. We have in addition added a calligraphic font to signify the random process itself, which is not just a vector of random variables like the past state. Further we would like to emphasize that AIS is indeed computed between a past state vector random variable ($Y_{<t}$) and a future individual random variable (Y_t). Thus, it is indeed intended that both regular and bold font versions of Y appear in the same formula, e.g. in the defining equation 1.
Where there are matrix-valued variables, we now use the double underline notation for the Matrices.

- n page 6, the authors state that "with this method, we are also able to handle any potential bias introduced by the wavelet filtering of the surrogate data." What does this mean? Could you elaborate?

Reply: We agree with the reviewer that the statement " handle any potential bias introduced by the wavelet filtering of the surrogate data" was misunderstandable. We now describe more clearly how our approach protects against false positive results in statistical testing.

- Page 7: Should AIS' be indexed by j? It depends on the wavelet scale, if I understand correctly.

Reply: This is correct, we have added the j in page 7.

- Paragraph after Eq. 9: should it be Fig 2C, instead of 2A?

Reply: We thank the reviewer for catching this error.

- Example III: the paragraph after Eq. 9 says that w_1,w_2 "are the natural frequencies of the oscillators which were set to 0.8 and 0.9." However, later it says that the process "oscillated around 8 Hz." How are these related? And what's the sampling rate of the process?

Reply: We changed the text to reflect that w_1 and w_2 are the parameters governing the frequencies, and not the frequencies themselves. We also now note the sampling rate of the process now (500Hz).

- Page 10: Remove the "0.0.1" numbering in the section header (same with "0.0.2" in page 16).
  Reply: We thank the reviewer for catching this error.

- Page 11: instead of "model squared," it would be more consistent with statistical literature to call it "quadratic model."
  We have changed the term "model squared" with "quadratic model"

- Page 11: I suspect there's something missing after "we report the full modeling and model comparison results in..."
  Reply: We thank the reviewer for catching this error.

- Page 16 and Fig. 11: I suspect something may have been misplaced in the figure, because the BFs reported in the text do not match those in the figure. For example, the first reported BF is "BF= 48530.5 (see Fig 11, panel A, top row)," while that panel has BF = 1.380.
  Reply: This is indeed true. The BF of panel A and panel B has been swapped. We have now corrected Figure 11 (which is Figure 6 with the new arrangement).

- How are the trials from both ferrets pooled together for analysis?

Reply: The spectral AIS was computed for each animal and experiment (if this was meant by trials) separately. Data from the 8 second sections (if this was meant by trials) of data in an individual experiment where entered jointly in the embedding space for neighbour searches in the computation of the AIS measure.

Have the authors made all data and (if applicable) computational code underlying the findings in their manuscript fully available?

Reply: We have now made a Dryad repository with the data. We will provide the URL as soon as the submission to the Dryad is completed and the link is available.
